# Awareness of Coronary Artery Disease Risk Factors Among the Population of Taif City, Saudi Arabia

**DOI:** 10.7759/cureus.30084

**Published:** 2022-10-08

**Authors:** Walid Abukhudair, Mohammed A Alosaimi, Bandar A Alghamdi, Riyadh A Alharthi, Ahood A Mahjari, Ibrahim A Albalawi, Reemah F Alqahtani

**Affiliations:** 1 Cardiac Surgery, King Fahad Armed Force Hospital, Jeddah, SAU; 2 Faculty of Medicine, Taif University, Taif, SAU; 3 Medicine, Najran University, Najran, SAU; 4 Faculty of Medicine, Tabuk University, Tabuk, SAU; 5 Faculty of Medicine, King Khalid University, Abha, SAU

**Keywords:** taif city saudi arabia, risk factors cardiovascular diseases, coronary artery disease, awareness, prevalence

## Abstract

Background: Coronary artery disease (CAD) is responsible for significant mortality and morbidity among patients. Many factors are associated with the increasing prevalence of CAD in a population, including diet and lifestyle, physical inactivity, high cholesterol levels, and others.

Objectives: The purpose of this study is to assess the awareness level and knowledge about CAD risk factors and its prevalence among the general population in Taif city, Saudi Arabia.

Methods: This study was a community-based cross-sectional descriptive study carried out from August 2022 to September 2022 by an online questionnaire previously validated in published studies and then distributed via different social media platforms to assess participants' knowledge of risk factors for CAD. The survey included questions about socio-demographic data and risk factors of cardiovascular diseases and their prevalence.

Results: A total of 2439 participants met the inclusion criteria and finally enrolled in the current study. About 1671 (68.5%) were found to have good awareness levels, 718 (29.4%) of the participants were considered to be having fair awareness levels, and only 50 (2.1%) of the participants were with poor awareness levels.

Conclusion: Most participants have a good level of knowledge and awareness about CAD. Few knowledge gaps were regarding certain factors, including age, gender, and family history of CAD. High educational level and age were found to be linked with a higher level of knowledge regarding CAD.

## Introduction

Coronary artery disease (CAD) accounts for more than 4.5 million deaths worldwide. Developing countries continue to experience rapid increases in CAD risk factors and mortality, despite recent declines in developed countries [1]. In developing countries, demographic and lifestyle changes are causing an “epidemiological transition” from infectious and perinatal diseases to non-communicable diseases such as CAD. There are many factors associated with the prevalence of CAD in a population, including diet and lifestyle. Lifestyle measures are, therefore, essential for the primary prevention of cardiovascular diseases [2]. It has been shown that angiotensin-converting enzyme (ACE) inhibitors therapy has no advantage over conventional management in a lower-risk population, which emphasizes the importance of lifestyle modification for this population [3].

Consumption of fats and sugars has increased due to the globalization of diet, which is associated with an increased risk of coronary artery disease. Living in an urban area appears to be associated with a higher prevalence of risk factors for CAD. Compared with rural dwellers, urbanites tend to be more sedentary and consume more fat and energy-dense foods. Certain parts of the world are also experiencing a rise in tobacco use due to urbanization. Atherosclerotic plaques in coronary arteries are associated with such lifestyle changes. There is a rapid urbanization trend in many developing countries. CAD is also strongly influenced by genetic factors, and in the past decade, significant progress has been made in this area. Several genome-wide association studies involving more than 200,000 people were conducted, as well as bioinformatics approaches, including 1,000 genomes imputation, expression quantitative trait locus analyses, and interrogation of Encyclopaedia of DNA Elements, Roadmap, and other databases [4].

Several countries, including Saudi Arabia (KSA), have seen an increase in obesity, high cholesterol, and metabolic disorders among adolescents and young adults in the last few decades. Furthermore, unhealthy lifestyle habits are responsible for 78% of all deaths due to heart disease, cancer, and diabetes. As a result of the frequent consumption of fast food, Saudis have been reported to be physically inactive and to consume unbalanced diets high in fat, saturated fats, sodium, and carbohydrates, and low in dietary fiber [5]. It includes pizza, shawarma, French fries, hamburgers, chicken nuggets, and other prepared foods bought outside the home [6]. According to a previous study investigating the relationship between fast-food consumption and obesity, KSA has a 5.5% CAD prevalence, midway between other countries. There are several classical CAD risk factors in the Saudi population, including older age, male gender, overweight, hypertension, smoking, diabetes mellitus, hypertriglyceridemia, and hypercholesterolemia. A significant role is also played by metabolic syndrome in the development of CAD in this group of individuals. Therefore, metabolic syndrome must be managed, as well as lifestyle changes to reduce modifiable risk factors for CAD. 

Studies on the risks and prevalence of CAD have been conducted for different cities in Saudi Arabia using cross-sectional methods, like studies in Qassim, Jeddah, Dawadmi, and Al-Qunfudah revealed that a large portion of the disease is associated with the diet of the population [7-11]. The sudden switch in dietary behavior from traditional to fast food severely impaired cardiovascular functions, causing them to become abnormal. The young population of Saudi Arabia is undergoing significant economic changes that have translated into the adoption of a Western dietary lifestyle. This has led to a significant increase in the burden of CVD. There is, however, a lack of adequate baseline knowledge of CAD risk factors. As public awareness increases, personal awareness and actions to reduce CVD risk increase too. Health promotion behaviors have been associated with the understanding of heart disease.

There is a lack of knowledge regarding the risk factors for CAD is evident among the population in Taif, Saudi Arabia. Therefore, our study includes an analysis of population awareness of CAD risk factors (including smoking, lack of physical activity, fast food and soft drink intake, being old, male, obese, and anxiety-prone, history of diabetes mellitus, high cholesterol, and high blood pressure, as well as a family history of CAD) [12] in Taif. 

## Materials and methods

Study design

This study was a community-based cross-sectional descriptive study conducted in Taif city, Kingdom of Saudi Arabia, from August 2022 to September 2022. 

Study population and sampling methodology

In this study, the general population residing in Taif city, Saudi Arabia who agreed to participate in the study were included. Any residents outside Taif city were excluded. Data were collected through a previously validated online questionnaire in a published study by Odah et al. [11]. It was formulated in Arabic, was completed using Google Documents, and distributed electronically via social media applications. There was a total of 3,680 who participate in the study, we ensured that the participants selected for the study belong to different parts of the city at random.

The questionnaire covered the following sections: A) The participants’ socio-demographic data, including age in years, gender, nationality, education, marital status, and employment status. B) Level of participants’ knowledge and awareness about CAD risk factors. C) Prevalence of risk factors for CAD, the questionnaire included 29-item, close-ended questions. Participants were asked to respond to knowledge items as either yes or no. Incorrect responses were given a score of zero, and correct answers were assigned a score of one.

Data analysis

SPSS software, version 23 (IBM Corp., Armonk, NY, USA) was used for data entry and analysis. Descriptive statistics such as mean score and standard deviation, as well as frequency and percentages of all independent variables, were used. Responses were scored by frequency and percentage, then converted to percentage mean scores and transformed into qualitative data. Analytic statistics were applied. The Chi-square test of independence or Fischer exact test was used for the analysis of qualitative data. Significance was considered at a p-value < 0.05.

Ethical considerations

Ethical approval was provided by the Institutional Review Board (IRB) of King Fahad Armed Forces Hospital, Jeddah (No-2022-45- REC 526). Consent was obtained electronically from all participants after the study aims were explained.

## Results

Socio-demographics data

Out of a total of 3,680 participants, 1,241 were excluded (did not meet the inclusion criteria), and 2,439 of the participants met the criteria and were included in the analysis. Table 1 shows that most of the participants, 1,126 (46.2%), were within the age group of 19-30 years, and 455 (18.7%) were within the age group of 41-50 years. Four hundred and twenty-seven (17.5%) were within the age group of 31-40 years, 228 (9.3%) were less than 18 years old, and 203 (8.3%) were more than 50 years old. About 1,457 (59.7%) were females, and 982 (40.3%) were males. In regard to the educational level, most of the participants, 1,416 (58.1%), had a postgraduate degree, 870 (35.7%) were of secondary educational level, 91 (3.7%) of the participants had an intermediate educational level, whereas 47 (1.9%) had primary education level, and 15 (0.6%) were found to be illiterate. About 1,241 (50.9%) of the participants were single, 1,028 (42.1%) were married, 134 (5.5%) were divorced, and 36 (1.5%) were widowed. Nine hundred and eighty-seven (987) (40.5%) of the participants were students, 766 (31.4%) were employees, 538 (22.1%) were unemployed, and 148 (6.1%) were retired.

**Table 1:** Socio-demographic characteristics of the study participants (n=2,439)

Variable	Category	Frequency	Percent
Age (years)	< 18	228	9.3%
	19-30	1126	46.2%
	31-40	427	17.5%
	41-50	455	18.7%
	> 50	203	8.3%
Gender	Male	982	40.3%
	Female	1457	59.7%
Educational level	Illiterate	15	0.6%
	Primary	47	1.9%
	Intermediate	91	3.7%
	Secondary	870	35.7%
	Postgraduate	1416	58.1%
Marital status	Single	1241	50.9%
	Married	1028	42.1%
	Divorced	134	5.5%
	Widowed	36	1.5%
Employment status	Student	987	40.5%
	Employee	766	31.4%
	Unemployed	538	22.1%
	Retired	148	6.1%

Awareness of risk factors for coronary artery disease

**Figure 1:**
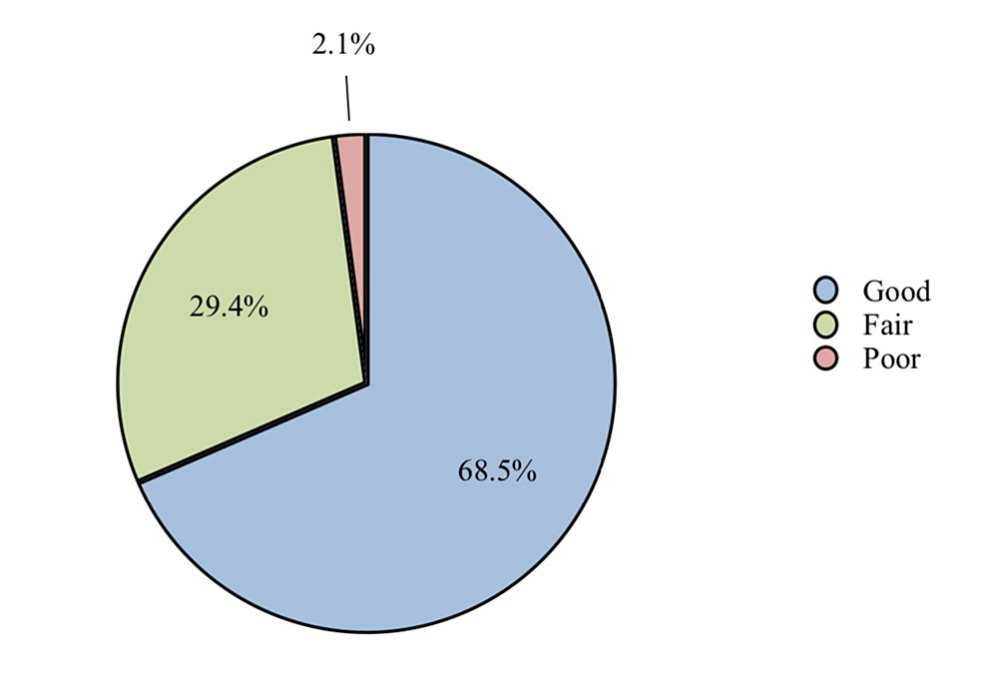
Level of awareness about risk factors for coronary artery disease

Out of a total score of 12, the mean awareness score was 9.3 ± 2.04 (Range 0-12). Figure 1 shows that the majority of respondents (1,671, 68.5%) have good awareness levels, 718 (29.4%) of the participants were considered to have fair awareness levels, and only 50 (2.1%) of the participants were with poor awareness levels.

Smokers are more likely to develop cardiovascular diseases. Table 2 showed as reported by 2,236 (91.7%) participants are smokers, whereas 203 (8.3%) did not think smoking is a risk factor for cardiovascular disease. The incidence of cardiovascular diseases increases with a residential lifestyle with a lack of exercise for five consecutive days. This statement was agreed on by 2,016 (82.7%), and 423 (17.3%) did not agree with the same statement. The majority (2,158, 88.5%) of the participants think eating fast foods increases the risk of cardiovascular diseases and 281 (11.5%) do not. Soft drinks were believed to be a risk factor for cardiovascular disease by 1,865 (76.5%). About 1,452 (59.5%) of the participants think that age is linked to cardiovascular disease, and 987 (40.5%) do not. More than half of the participants (1,283, 52.6%) think that a family member with a cardiovascular disease increases the risk of cardiovascular disease in the family. High cholesterol levels increase cardiovascular disease risk, as agreed on by 2,163 (88.7%) of the participants, and 1,873 (76.8%) of the participants think having high blood sugar levels increases the risk of cardiovascular diseases. Obesity is believed by 2,218 (90.9%) of the participants to be one factor that increases the risk of cardiovascular disease. About 2,066 (84.7%) of the participants think that anxiety and stress increase cardiovascular disease risk. About 1,119 (45.9%) think that males are more susceptible to cardiovascular diseases than females, and 2,153 (88.3%) of the participants think high blood pressure increases the risk of cardiovascular diseases. 

**Table 2:** Awareness of risk factors for coronary artery disease

Question	Yes	No
	N (%)	
1. Do you think that smokers are more likely to have cardiovascular disease?	2236 (91.7)	203 (8.3)
2. Do you think that not exercising at least 30 minutes of walking daily for 5 days increases the incidence of cardiovascular disease?	2016 (82.7)	423 (17.3)
3. Do you think that eating fast food increases the risk of cardiovascular disease?	2158 (88.5)	281 (11.5)
4. Do you think that soft drinks increase the risk of cardiovascular disease?	1865 (76.5)	574 (23.5)
5. Do you think that age is linked to cardiovascular disease?	1452 (59.5)	987 (40.5)
6. Do you think that having a family member with cardiovascular disease increases your risk of cardiovascular disease?	1283 (52.6)	1156 (47.40
7. Do you think that high cholesterol in the blood increases the risk of cardiovascular disease?	2163 (88.7)	276 (11.3)
8. Do you think that high blood sugar (diabetes) increases the risk of cardiovascular disease?	1873 (76.8)	566 (23.2)
9. Do you think that obesity increases the risk of cardiovascular disease?	2218 (90.9)	221 (9.1)
10. Do you think that anxiety and stress increase the risk of cardiovascular disease?	2066 (84.7)	373 (15.3)
11. Do you think that males are more susceptible to cardiovascular disease than females?	1119 (45.9)	1320 (54.1)
12. Do you think that high blood pressure increases the risk of cardiovascular disease?	2153 (88.3)	286 (11.7)

Prevalence of risk factors for coronary artery disease

**Figure 2:**
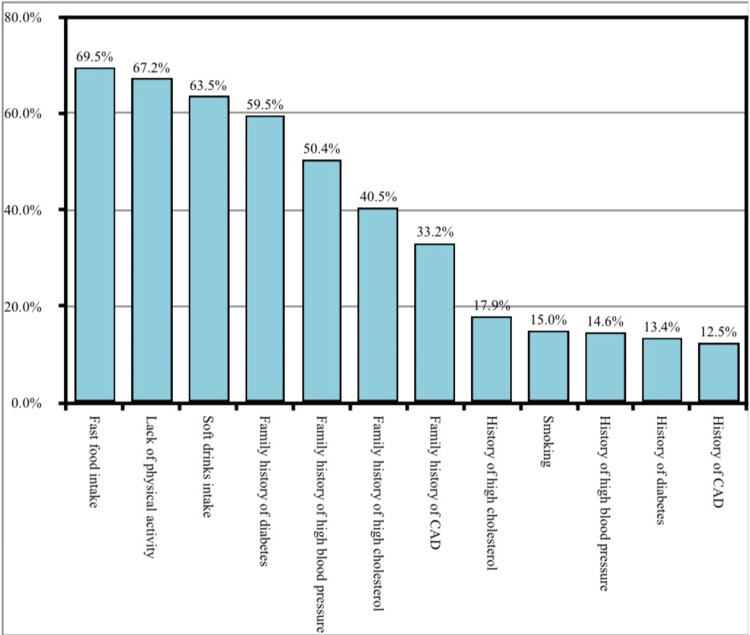
Prevalence of risk factors for CAD among the study participants

Figure 2 shows the prevalence of risk factors for cardiovascular diseases; 69.5% of the participants were taking fast food and 67.6% of the participants lacked physical activity. Soft drink intake was reported by 63.3% of the participants, 59.5% of the participants were having a family history of diabetes, 50.4% were with a family history of high blood pressure, 40.5% were with a family history of high cholesterol, 32.2% were with a family history of coronary artery disease, 15% of the participants were smokers, 14.6% of the participants had a history of high blood pressure, 13.4% of the participants had a history of diabetes and 12.5% of the participants had a history of CAD.

Factors associated with awareness of risk factors for CAD

Table 3 shows that age was found to be significantly associated with the level of awareness regarding risk factors of CAD (p-value ˂ 0.001), with the age group of more than 50 years old having a higher level of awareness than other groups. Gender was not found to be significantly associated with awareness level about cardiovascular disease (p-value = 0.066). A statistically significant association was found between educational level and awareness level about the risk factors of CAD (p-value ˂ 0.001), with postgraduate and primary educational levels having a higher level of awareness than other groups. Marital status was found to be significantly associated with the level of awareness about the risk factors for CAD (p-value ˂ 0.001), as being married was linked to higher awareness levels than other marital status groups. Employment status was not significantly associated with the level of awareness about the risk factors for CAD (p-value = 0.069).

**Table 3:** Factors associated with awareness of risk factors for CAD.

Variable	Category	Level of Awareness			P value
		Poor	Fair	Good	
		N (%)			
Age (years)	< 18	11 (4.8)	91 (39.9)	126 (55.3)	< 0.001
	19-30	23 (2)	359 (31.9)	744 (66.1)	
	31-40	6 (1.4)	124 (29)	297 (69.6)	
	41-50	6 (1.3)	103 (22.6)	346 (76)	
	> 50	4 (2)	41 (20.2)	158 (77.8)	
Gender	Male	25 (2.5)	308 (31.4)	649 (66.1)	0.066
	Female	25 (1.7)	410 (28.1)	1022 (70.1)	
Educational level	Illiterate	1 (6.7)	5 (33.3)	9 (60)	< 0.001
	Primary	5 (10.6)	7 (14.9)	35 (74.5)	
	Intermediate	7 (7.7)	26 (28.6)	58 (63.7)	
	Secondary	17 (2)	284 (32.6)	569 (65.4)	
	Postgraduate	20 (1.4)	396 (28)	1000 (70.6)	
Marital status	Single	33 (2.7)	410 (33)	798 (64.3)	< 0.001
	Married	11 (1.1)	264 (25.7)	753 (73.2)	
	Divorced	3 (2.2)	33 (24.6)	98 (73.2)	
	Widowed	3 (8.3)	11 (30.6)	22 (61.1)	
Employment status	Student	19 (1.9)	312 (31.6)	656 (66.5)	0.069
	Employee	12 (1.6)	201 (26.2)	553 (72.2)	
	Unemployed	13 (2.4)	166 (30.9)	359 (66.7)	
	Retired	6 (4.1)	39 (26.4)	103 (69.6)	

## Discussion

Evaluation and assessment of the knowledge and awareness levels about cardiovascular diseases among the population will result in the determination of knowledge gaps which could be addressed with suitable intervention methods accordingly, and also greater levels of knowledge about coronary artery diseases allow individuals to correctly assess their risk of having coronary artery disease [13,14]. This study aimed to determine whether Saudis are aware of CAD risk factors and the prevalence of risk factors that put them at risk for developing CAD. About less than half of the participants (46.2%) were within the age group of 19-30 years old. More than half (59.7%) were females, and (40.3%) were males. Regarding the educational level, most participants (58.1%) had a postgraduate degree. Half (50.9%) of the participants were single. Less than half (40.5%) of the participants were students.

Out of a total score of 12, the mean awareness score was 9.3. About (68.5%) were found to be having good awareness levels, less than one-third (29.4%) of the participants were considered to be having fair awareness levels, and only 2.1% of the participants were with poor awareness levels. This was found to be contradictory to the study conducted by Ammouri et al. in which 60.5% of the participants had adequate knowledge scores [15].

Regarding knowledge about the risk factors of CAD, smokers are more likely to develop cardiovascular diseases, as reported by the vast majority (91.7%) of the participants. This was found to be consistent with the study by Akintunde et al., in which (79.6%) of the participants were oriented about the increased risk of CAD with smoking [16]. Cardiovascular disease incidence increases with a residential lifestyle with a lack of exercise for five consecutive days; this statement was agreed on by the majority (82.7%) of the participants. The vast majority (88.5%) of the participants think eating fast foods increases the risk of cardiovascular diseases. Soft drinks were believed to be a risk factor for cardiovascular diseases by (76.5%) of the participants. More than half (59.5%) of the participants think that age is linked to cardiovascular diseases. About half (52.6%) of the participants think that a family member with a cardiovascular disease increases the risk of cardiovascular disease in the family. The vast majority of the participants (88.7%) think that high cholesterol level increases the risk of cardiovascular disease. More than two-thirds (76.8%) of the participants think that having high blood levels of sugars increases the risk of cardiovascular diseases. Obesity was believed by the majority (90.9%) of the participants to be one factor that increases the risk of cardiovascular disease. Most (84.7%) of the participants think that anxiety and stress increase cardiovascular disease risk. Slightly less than half (45.9%) think that males are more susceptible to cardiovascular diseases than females. High blood pressure increases the risk of cardiovascular diseases, as reported by (88.3%) of the participants. Similar findings were reported in the parallel study by Awad and Al-Nafisi, in which most participants were aware that smoking, obesity, unhealthy diet, and physical inactivity were associated with an increased risk of coronary artery disease [17].

Concerning the prevalence of risk factors for cardiovascular diseases, the most prevalent risk factor was the consumption of fast food, followed by physical inactivity. The majority (69.5%) of the participants were taking fast food and two-thirds (67.6%) of the participants lacked physical activity. Soft drink intake was reported by (63.3%) of the participants, (59.5%) of the participants were having a family history of diabetes, and (50.4%) were with a family history of high blood pressure. This was consistent with the congruent study by Sekhri et al., in which the most prevalent risk factors for CAD among participants were diabetes mellitus, hypertension, and high cholesterol levels [18].

A significant association was found between age and level of awareness regarding risk factors of CAD, with the age group of 41-50 and more than 50 years old having a higher awareness level than other groups. Similar findings were reported in the parallel study by Mugamammi et al., in which the middle-aged group was linked with a higher knowledge level of CAD [19]. Marital status was significantly associated with awareness of the risk factors for CAD. Being married was linked to higher awareness levels than other marital status groups. Statistically significant association was found between educational level and awareness level about CAD risk factors. Participants with postgraduate and primary educational levels had a higher awareness level than other groups. This was consistent with the findings of Alruway’s study in which university educational level was associated with higher knowledge levels about CAD [12].

Study limitations

We are aware of a few study limitations that should be addressed. First, the study collected data using an online questionnaire, which may affect its validity if the answers were researched. The population of Taif city of Saudi Arabia is not high enough to represent the population of Saudi Arabia; hence, the results cannot be generalized to the rest of the kingdom.

## Conclusions

Most participants have a good level of knowledge and awareness about coronary artery diseases. A few knowledge gaps regarding certain factors, including age, gender, and family history of CAD exist. High educational level and age were linked to a higher level of knowledge regarding CAD. Knowledge gaps should be addressed through health education programs and augmentation of the role of media in more distribution of knowledge.
